# Significant association between intracranial volume and verbal intellectual abilities in patients with schizophrenia and a history of birth asphyxia

**DOI:** 10.1017/S0033291721000489

**Published:** 2022-11

**Authors:** Laura Anne Wortinger, Kjetil Nordbø Jørgensen, Claudia Barth, Stener Nerland, Runar Elle Smelror, Anja Vaskinn, Torill Ueland, Ole A. Andreassen, Ingrid Agartz

**Affiliations:** 1 Department of Psychiatric Research, Diakonhjemmet Hospital, Oslo, Norway; 2 NORMENT, Institute of Clinical Medicine, University of Oslo, Oslo, Norway; 3 Division of Mental Health and Addiction, NORMENT, Oslo University Hospital, Oslo, Norway; 4 Department of Psychology, University of Oslo, Oslo, Norway; 5 Department of Clinical Neuroscience, Centre for Psychiatric Research, Karolinska Institutet, Stockholm, Sweden

**Keywords:** Asphyxia, bipolar disorders, intracranial volume, IQ, schizophrenia

## Abstract

**Background:**

The etiology of schizophrenia (SZ) is proposed to include an interplay between a genetic risk for disease development and the biological environment of pregnancy and birth, where early adversities may contribute to the poorer developmental outcome. We investigated whether a history of birth asphyxia (ASP) moderates the relationship between intracranial volume (ICV) and intelligence in SZ, bipolar disorder (BD) and healthy controls (HC).

**Methods:**

Two hundred seventy-nine adult patients (18–42 years) on the SZ and BD spectrums and 216 HC were evaluated for ASP based on information from the Medical Birth Registry of Norway. Participants underwent structural magnetic resonance imaging (MRI) to estimate ICV and intelligence quotient (IQ) assessment using the Wechsler Abbreviated Scale of Intelligence (WASI). Multiple linear regressions were used for analyses.

**Results:**

We found a significant three-way interaction (ICV × ASP × diagnosis) on the outcome variable, IQ, indicating that the correlation between ICV and IQ was stronger in patients with SZ who experienced ASP compared to SZ patients without ASP. This moderation by ASP was not found in BD or HC groups. In patients with SZ, the interaction between ICV and a history of the ASP was specifically related to the verbal subcomponent of IQ as measured by WASI.

**Conclusions:**

The significant positive association between ICV and IQ in patients with SZ who had experienced ASP might indicate abnormal neurodevelopment. Our findings give support for ICV together with verbal intellectual abilities as clinically relevant markers that can be added to prediction tools to enhance evaluations of SZ risk.

## Introduction

Schizophrenia (SZ) is a complex mental disorder that occurs globally with an international prevalence estimate of 0.33–0.75% (NIMH, 2018). A recent study reports that the most significant genetic variants that contribute to SZ risk were found to interact with the biological environment of pregnancy and birth (Ursini et al., 2018). Specifically, SZ risk-associated genes differentially expressed in placentae were significantly enriched for pathways related to metabolic and cellular stress and hypoxia (Ursini et al., 2018). These findings suggest that part of the genetic predisposition for SZ may relate to intolerance to hypoxic stress.

Worldwide, newborn infants suffer permanent brain damage or death as a result of hypoxia (i.e. deficiency in the amount of oxygen reaching tissues) caused by birth asphyxia (ASP) – a condition where the newborn has been deprived of oxygen before or around the time of birth (i.e. peripartum period). Neonatal hypoxic-ischemic encephalopathy (HIE) is a profound systemic and neurological consequence of decreased blood flow and/or oxygen to the brain and the sequelae of ASP. Not all incidences of ASP end in a diagnosis of HIE, which can take a day or more to conclude, and a diagnosis of HIE is graded as mild, moderate or severe. Most infants with neonatal HIE and hypoxic-ischemic insult evident on magnetic resonance imaging (MRI) have an injury from the immediate peripartum period, and not from long-term antenatal damage (Inder & Volpe, 2018). Causes of ASP are complex and often relate to uterine rupture, placental and umbilical cord complications (abruptio placentae, cord prolapse) and infection (Inder & Volpe, 2018). In Sweden, the incidence of ASP was 7.2 per 1000 live births, wherein ASP with HIE was 1.8 per 1000 live births (Thornberg, Thiringer, Odeback, & Milsom, 1995), an HIE rate similar to other developed countries (Kurinczuk, White-Koning, & Badawi, 2010). Empirical studies show that the prevalence of ASP is similar across psychiatric patient and healthy groups (Ursini et al., 2018; Wortinger et al., 2020). A population study from Sweden found that ASP increased the risk for SZ (odds ratio 4.4) (Dalman et al., 2001). The perinatal period is a sensitive time in brain development, and a hypoxic insult due to ASP can have long-term consequences on brain function (Ducsay et al., 2018; Volpe, 2012).

The neurodevelopmental hypothesis of SZ suggests a vulnerability for serious obstetric complications that interacts with genetic factors and influences the development of SZ (Birnbaum & Weinberger, 2017; Stilo & Murray, 2019). Empirical research linking greater disability and altered neuronal growth in patients with SZ who have a history of the ASP has been scarce (Davies et al., 2020; Radua et al., 2018). However, we recently reported lower intelligence quotient (IQ) across adult patients with SZ and bipolar disorder (BD) and healthy controls (HC) who experienced more than one serious obstetric complication, wherein ASP was one of the complications registered in 81% of all cases (Wortinger et al., 2019). BD is a severe mental disorder characterized by affective episodes and psychotic symptoms present in 60% of cases. BD is considered as part of the psychosis continuum, with overlapping clinical characteristics, genetic risk and pharmacological treatment as SZ (Vieta et al., 2018). Additionally, we found that adult height was lower in both patients with SZ and HC who experienced ASP (Wortinger et al., 2020). Birth and pregnancy information were prospectively obtained from the Medical Birth Registry of Norway in both studies (Wortinger et al., 2019, 2020). These findings might not only indicate greater intellectual deficits in those who experience ASP, but also gross alterations to normal developmental trajectories.

MRI derived intracranial volume (ICV) estimates strongly correlate with head circumference measures, *r* = 0.70 (Hshieh et al., 2016) and can be used as a proxy for both head size and a marker of early brain development. ICV reaches about 90% of its full size around the age of 5 years, which suggests brain growth during the early years determines cranium size (Gilmore, Knickmeyer, & Gao, 2018; Sgouros, Goldin, Hockley, Wake, & Natarajan, 1999; Woods, 1998). ICV matures while the brain goes through additional structural and functional development until mid-to-late adolescence (Mills et al., 2016; Sgouros et al., 1999), making early childhood and adolescent periods particularly sensitive to injury.

A relatively smaller ICV is commonly reported for patients with SZ compared to HC (Gurholt et al., 2020; Haijma et al., 2013; Hulshoff Pol et al., 2012; Okada et al., 2016; van Erp et al., 2016). We found smaller ICV across adult patients with SZ and BD and HC who experienced ASP compared to those who did not experience ASP (Wortinger et al., 2020). In another study, offspring of patients with SZ had smaller ICV than both offspring of patients with BD and HC, but both offspring of patients with SZ and BD had lower IQs than HC offspring (van Haren et al., 2020). These findings imply that familial risk for SZ has a stronger association with impeded early brain development than the familial risk for BD.

Brain volume and intelligence positively correlate in the general population, *r* = 0.30 (Cox, Ritchie, Fawns-Ritchie, Tucker-Drob, & Deary, 2019; Gignac & Bates, 2017). It has been suggested that this relationship is stronger in SZ (Jensen et al., 2019; Rais et al., 2012; van Haren et al., 2020). Genome-wide association studies suggest that a large fraction of the genomic risk for SZ influences both intelligence and ICV (Smeland et al., 2018, 2019). If this genomic risk relates to a vulnerability to hypoxic stress, individuals who experience ASP at birth and later developmental SZ could have altered neurodevelopmental trajectories already from birth. Depending on the duration of oxygen insufficiencies to the brain, ASP may relate to varying degrees of neural harm in the offspring. The degree of neural harm caused by ASP might be represented by a greater relationship between ICV and IQ in SZ patients because of the susceptibility of these factors already present in the disorder. On this background, we hypothesized that a greater positive relationship between ICV and IQ exists, specifically among patients with SZ who have a history of the ASP. Effects of ASP on both IQ and ICV have been demonstrated in previous studies, but the extent to which these are linked in SZ has not been investigated.

In this study, we combined the data of two previous studies (Wortinger et al., 2019, 2020) and investigated whether a history of the ASP interacts with the relationship between adult ICV and IQ in patients on the SZ and BD spectrums and HC. The aims of this study were (1) measure the prevalence of ASP across groups; (2) compare IQ and ICV, after controlling for the effects of ASP, between groups to investigate whether we can identify evidence of abnormal neurodevelopment; (3) examine the relationships between IQ, ICV and ASP between SZ, BD and HC; (4) explore which subcomponents of IQ (verbal or performance) as measured by WASI are most related to ICV and ASP.

## Methods

This study is part of the Thematically Organized Psychosis (TOP) study, which is a thematic research effort focused on disease mechanisms of psychotic disorders and is the main study protocol at the Norwegian Center for Mental Disorders Research (NORMENT, Oslo, Norway). Adult patients were recruited from psychiatric units (outpatient and inpatient) of public hospitals in the Oslo region. The HC were randomly selected from the national population register in the same catchment area as the patients. After a complete description of the study, all participants gave written informed consent. The study was conducted in accordance with the Helsinki Declaration and the South East Regional Committee for Research Ethics (REC South-East). The Norwegian Data Inspectorate approved the study.

### Participants

All patients underwent a thorough clinical investigation by trained psychologists and physicians. Clinical diagnoses were assessed using the Structured Clinical Interview for the Diagnostic and Statistical Manual of Mental Disorders, 4th Edition (DSM-IV), Axis 1 disorder (SCID-I) module A-E (Spitzer, Williams, Gibbon, & First, 1988). The psychosocial function was assessed with the Global Assessment of Functioning scale, split version (GAF; Pedersen Hagtvet, & Karterud, 2007). Current psychotic symptoms were rated by the use of the Positive and Negative Syndrome Scale (PANSS; Kay, Fiszbein, & Opler, 1987). The Alcohol Use Disorders Identification Test (AUDIT) (Saunders, Aasland, Babor, de la Fuente, & Grant, 1993) and Drug Use Disorders Identification Test (DUDIT) (Berman, Bergman, Palmstierna, & Schlyter, 2005) were used to evaluate alcohol and drug use in patients. HC were interviewed by trained research assistants and examined with the Primary Care Evaluation of Mental Disorders (Prime-MD) to ensure no current or previous psychiatric disorders (Spitzer et al., 1994).

Exclusion criteria for both patients and HC were hospitalized for previous moderate or severe head injury, neurological disorder, medical conditions thought to interfere with brain function and age outside the range of 18–65 years. All MRI scans go through a thorough screening by a neuroradiologist and a graded scheme in the MR lab, and if pathology is detected in the MR images, the participant is excluded. Additional exclusion criteria for HC were current or previous somatic illness and substance misuse disorders or dependency within the last 6 months. HC were also excluded if they or a first-degree relative had a lifetime history of severe psychiatric disorder.

For the current study, participants were included if they had a birth registry, IQ and MRI data. No matching procedures were performed to ensure similarity between the groups in, e.g. age, sex or education. The total subject sample (*N* = 495, age range: 18–42 years) consisted of patients with a DSM-IV diagnosis within the *SZ spectrum*: SZ (DSM-IV 295.1, 295.3, 295.6 and 295.9; *n* = 93), schizophreniform disorder (DSM-IV 295.4; *n* = 15), schizoaffective disorder (DSM-IV 295.7; *n* = 10) or psychosis not otherwise specified (DSM-IV 298.9; *n* = 53); or within the *BD spectrum*: bipolar I disorder (DSM-IV 296.0–7; *n* = 67), bipolar II disorder (DSM-IV 296.89; *n* = 35) or BD not otherwise specified (DSM-IV 296.80; *n* = 6); and HC (*n* = 216).

### Birth asphyxia

Birth data were collected from the Medical Birth Registry of Norway (MBRN; https://www.fhi.no/en/hn/health-registries/medical-birth-registry-of-norway) and assessed for the presence of ASP. ASP was defined as having had a report of a complication coded AS53 (asphyxia without other signs), AS54 (asphyxia with poor fetal sound), AS55 (asphyxia and discolored amniotic fluid), AS56 (asphyxia with poor fetal sound and discolored amniotic fluid) or AS61 (asphyxia) on the MBRN registry form, all of which state the presence of newborn ASP.

### IQ measure

The IQ was assessed with the Wechsler Abbreviated Scale of Intelligence (WASI) (Wechsler, 2007), a Norwegian version translated from the original WASI (Wechsler, 1999). The WASI provides a total IQ score as well as scores for verbal and performance IQ. The WASI was developed as a short measure based on the Wechsler Adult Intelligence Scale (WAIS), which is considered the full IQ test. The WASI provides a good estimate of intelligence that is similar to WAIS (Bosnes, 2009; McCrimmon & Smith, 2013). The WASI IQ score is derived from four tests (vocabulary, similarities, block design and matrix reasoning) comprised of measures of verbal and nonverbal intelligence. The vocabulary and similarities tests form the verbal IQ subcomponent, which is an estimate of an individual's overall verbal intellectual ability. The two nonverbal tests (block design and matrix reasoning) form the performance IQ subcomponent, which is an estimate of perceptual and visuospatial organization.

### Image acquisition and processing

All participants underwent MRI scanning on a 1.5 T Siemens Magnetom Sonata scanner (Siemens Medical Solutions, Erlangen, Germany) equipped with an eight-channel head coil. After a conventional three-plane localizer, two sagittal T1-weighted magnetization prepared rapid gradient echo (MPRAGE) volumes were acquired with the Siemens tfl3d1_ns pulse sequence (echo time,TE = 3.93 ms; repetition time, TR = 2730 ms; inversion time, TI = 1000 ms; flip angle = 7°; field-of-view, FOV = 24 cm; voxel size = 1.33 × 0.94 × 1 mm^3^; number of partitions = 160).

The FreeSurfer software package (version 5.3) was used to process images (Dale, Fischl, & Sereno, 1999; Fischl et al., 2002). FreeSurfer estimates ICV by using the relationship between the ICV and the linear transform to MNI305 space [talairach.xfm; Buckner et al. (2004)].

### Statistical analysis

#### Asp and demographic variables

Between-group comparisons were performed with one-way ANOVAs for continuous variables, and Pearson χ^2^ tests for categorical variables. Post hoc, pairwise group comparisons were performed in cases of significant main effects of group. Pearson correlations were calculated for verbal IQ and performance IQ in each group. The correlations were compared using multiple regression analysis, controlling for age and sex. Pearson correlations were also calculated for verbal IQ and negative symptoms in each group, a relationship often viewed as interrelated (Harvey, Koren, Reichenberg, & Bowie, 2006). The correlations were compared using multiple regression analysis, controlling for age and sex. Statistical analyses were performed using the Statistical Package of Social Sciences (SPSS) version 26 (IBM SPSS Inc.).

#### IQ and ICV between groups

IQ and ICV were compared between groups using ANCOVAs with the group as a fixed factor (*α* = 0.05). Age, sex and ASP were used as covariates. Post hoc, pairwise group comparisons were performed.

#### IQ and ICV relationship

IQ was added as a dependent variable in a multiple regression model to assess its relationship with ICV between groups and with the effects of ASP. To assess the effects of patient groups, BD and SZ were dummy coded with HC as a reference and entered, together with ICV and ASP, as independent variables. ICV and patient group interactions were added as BD-by-ICV and SZ-by-ICV. We used an interaction term with ASP to determine whether ASP moderated the relationship between ICV (ASP-by-ICV) and the outcome variable, IQ. Also, interaction terms with ASP were used to determine whether ASP interacted with the variable, group, that was driven by the contrast in associations between the two groups (BD-by-ASP, SZ-by-ASP) or whether ASP interacted with both group and ICV variables (BD-by-ASP-by-ICV and SZ-by-ASP-by-ICV) to predict IQ. Age and sex were included as covariates in the model.

#### IQ subcomponents and ICV relationships

Follow-up analyses were performed for both verbal IQ and performance IQ using the same regression model as the IQ analysis above.

## Results

### Asp and demographic variables

ASP was prevalent in 14.3% of our sample (Table 1). More patients with BD experienced ASP, compared to the SZ group, but the prevalence of ASP did not significantly differ between patient groups and HC. There was a larger proportion of females in the BD group compared to the other groups. HC were older and had more years of education than the patient groups. The SZ group acquired less education than the BD group. A greater degree of clinical symptoms and reduced general functioning was found in the SZ group using GAF and PANSS scores compared to the BD group (Table 2). There were no differences in AUDIT (Alcohol Use Disorders Identification Test) and DUDIT (Drug Use Disorders Identification Test) scores between patient groups.

**Table 1.** ASP, demographics, ICV and IQ between groups

Group	HC (*n* = 216)	BD (*n* = 108)	SZ (*n* = 171)			Pairwise					
				Main effect		SZ *v.* HC		BD *v.* HC		SZ *v.* BD	
				*F/*χ^2^ (df)	*p*		*p*		*p*		*p*
Birth asphyxia + /−	32/184 (14.8%)	22/86 (20.4%)	17/154 (9.9%)	5.93 (2, 495)	0.052		0.206		0.206	(*BD>SZ*)	**0**.**014**
Sex m/f (% female)	123/93 (43.1%)	47/61 (56.5%)	103/68 (39.8%)	7.98 (2, 495)	**0**.**019**		0.514	(*BD>HC*)	**0**.**023**	(*BD>SZ*)	**0**.**006**
	Mean (s.d.)	Mean (s.d.)	Mean (s.d.)			Mean difference (CI 95%)		Mean difference (CI 95%)		Mean difference (CI 95%)	
Age	29.33 (5.93)	28.06 (6.07)	26.72 (5.71)	9.39 (2, 492)	**<0**.**001**	−2.61 (−3.79, −1.43)	**<0**.**001**	−1.27 (−2.64, 0.09)	0.067	−1.34 (−2.76, 0.09)	0.065
Education	14.37 (2.34)	13.39 (2.21)	12.71 (2.49)	23.90 (2, 492)	**<0**.**001**	−1.66 (−2.13, −1.18)	**<0**.**001**	−0.98 (−1.53, −0.43)	0.**001**	−0.68 (−1.25, −0.11)	**0**.**020**
ICV (cm^3^) ^a^	1634.7 (135.5)	1636.0 (134.2)	1606 (136.1)	2.53 (2, 489)	0.081	−28.85 (−56.68, −1.02)	**0**.**042**	1.26 (−30.04, 32.56)	0.937	−30.10 (−62.80, 2.60)	0.071
IQ ^a^	114.28 (11.88)	109.75 (11.89)	105.53 (11.97)	25.37 (2, 489)	**<0**.**001**	−8.75 (−11.17, −6.33)	**<0**.**001**	−4.53 (−7.28, −1.77)	0.**001**	−4.22, (−7.12, −1.33)	**0**.**004**
Verbal IQ ^a^	110.06 (12.55)	108.13 (12.56)	102.76 (12.65)	16.07 (2, 489)	**<0**.**001**	−7.30 (−9.86, −4.74)	**<0**.**001**	−1.93 (−4.84, 0.98)	0.193	−5.37 (−8.43, −2.30)	**0**.**001**
Performance IQ ^a^	115.55 (12.14)	109.38 (12.14)	107.21 (12.23)	23.82 (2, 489)	**<0**.**001**	−8.34 (−10.81, −5.86)	**<0**.**001**	−6.17 (−8.98, −3.36)	**<0**.**001**	−2.17 (−5.13, 0.79)	0.150

s.d. (standard deviation); df (degrees of freedom); CI (confidence intervals); HC (healthy controls); BD (bipolar disorder spectrum); SZ (schizophrenia spectrum); IQ (intelligence quotient); ICV (intracranial volume).

Bold values denote statistical significance at the *p* < 0.05 level.

a Effect of a group, corrected for sex, age and birth asphyxia.

**Table 2.** Clinical information between patient groups

Group	BD (*n* = 108)	SZ (*n* = 171)	Main effect	
	Mean (s.d.)	Mean (s.d.)	*F* (df)	*p*
GAF symptoms	55.81 (11.14)	43.45 (12.15)	73.01 (1, 277)	**<0**.**001**
GAF function	53.85 (12.78)	45.35 (12.21)	30.94 (1, 277)	**<0**.**001**
PANSS total	46.74 (10.05)	60.56 (17.02)	57.27 (1, 271)	**<0**.**001**
AUDIT	9.34 (6.86)	8.06 (7.32)	1.09 (1, 160)	0.297
DUDIT	4.24 (8.04)	5.37 (8.66)	0.62 (1, 160)	0.432

s.d. (standard deviation); df (degrees of freedom); BD (bipolar disorder spectrum); SZ (schizophrenia spectrum).

Bold values denote statistical significance at the *p* < 0.05 level.

### IQ and ICV between groups

A marginally significant main effect of group was found for ICV (Table 1), after controlling for age, sex and ASP. Pairwise comparisons revealed significantly smaller ICV in the SZ group than in the HC and BD groups (albeit trend level in the SZ–BD comparison). There was no difference in the ICV of BD and HC groups.

A significant main effect of group was found for IQ, verbal IQ and performance IQ (Table 1), after controlling for age, sex and ASP. Pairwise comparisons revealed significantly lower IQs in the SZ and BD groups as compared with HC and in SZ compared to BD. Verbal IQ was lower in patients with SZ compared to both HC and BD groups, but verbal IQ did not differ between BD and HC groups. Performance IQ was significantly lower in both patient groups compared to HC, but it did not differ between BD and SZ groups.

The correlations between verbal IQ and performance IQ within each group were all significant (verbal IQ-performance IQ: *r*_HC_ = 0.37, *p* = <0.001; *r*_BD_ = 0.38, *p* = <0.001; *r*_SZ_ = 0.55, *p* = <0.001). In a multiple regression analysis with verbal IQ as the outcome variable, we found that the correlation between verbal IQ and performance IQ was significantly greater in the SZ group compared to HC (SZ × performance IQ, *t* = 2.11, *p* = 0.036), controlling for age and sex, which was not the case for the BD group.

The correlations between verbal IQ and negative symptoms within each group were significant (*r*_BD_ = −0.23, *p* = 0.018; *r*_SZ_ = −0.24, *p* = 0.002). In a multiple regression analysis with verbal IQ as the outcome variable, we found the correlation between verbal IQ and negative symptoms was significantly greater in both BD and SZ patients combined and with ASP compared to patients without ASP (ASP × negative symptoms, *t* = −2.60, *p* = 0.010), controlling for age and sex.

### IQ and ICV relationship

In the regression analysis, we found a significant main effect of ICV across diagnostic groups (larger ICV associated with higher IQ). The slope of the relationship between ICV and IQ in patient groups did not differ from HC. There was no main effect of ASP across the whole sample and a non-significant interaction between ICV and ASP. However, a significant interaction between ASP and the SZ group indicated that within the SZ group, specifically, patients with a history of the ASP had lower IQs than patients without a history of the ASP. Furthermore, a significant three-way interaction between ASP, ICV and SZ showed that the relationship between ICV and IQ differed by ASP in patients with SZ. Specifically, patients with SZ and ASP displayed a stronger relationship compared to patients with SZ, but without ASP (Supplemental materials: online Supplementary Fig. 1*A*; online Supplementary Table 3).

### IQ subcomponents and ICV relationships

#### Verbal IQ

**Fig. 1.**
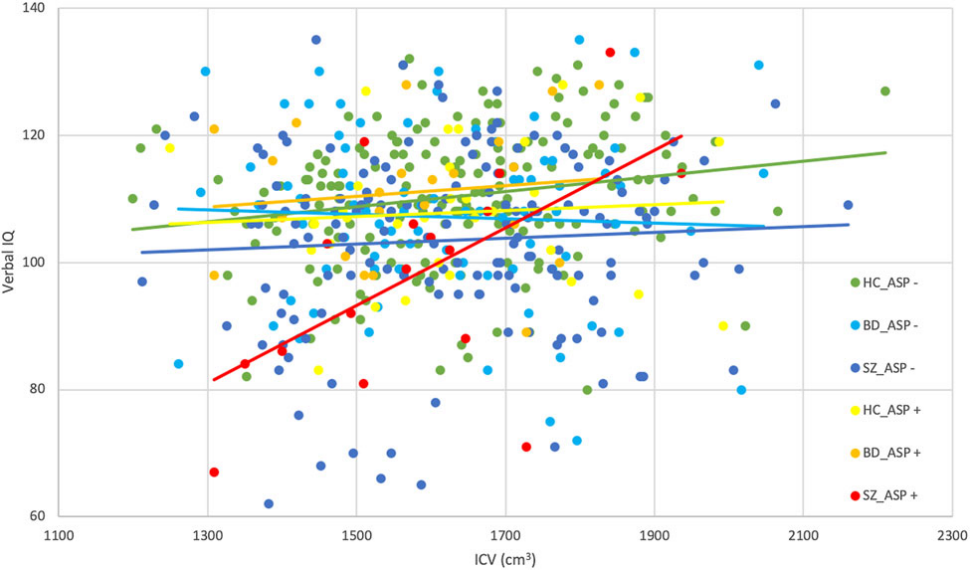
In the SZ group, the relationship between ICV and verbal IQ was significantly greater in the presence of ASP compared to when ASP was not present, which was not found in the other groups. SZ (schizophrenia spectrum); IQ (intelligence quotient); ICV (intracranial volume); ASP (birth asphyxia).

Similar to the model for IQ, we found a significant main effect of ICV across diagnostic groups in the model predicting verbal IQ. The slope of the relationship between ICV and verbal IQ in patient groups did not differ from HC. There was no main effect of ASP across the whole sample and a non-significant interaction between ICV and ASP. However, a significant interaction between ASP and the SZ group indicated that within the SZ group, specifically, patients with a history of the ASP had lower verbal IQ than patients without a history of the ASP. Furthermore, a significant three-way interaction between ASP, ICV and SZ showed that the relationship between ICV and verbal IQ differed by ASP in patients with SZ. Specifically, patients with SZ and ASP displayed a stronger relationship compared to patients with SZ, but without ASP (Fig. 1; Table 3).

**Table 3.** Multiple linear regression analyses by the level of IQ performance

	*B*	s.e.	*β*	*t*	*p*	*F*	df	*p*	adj. *R*^2^
**IQ**									
Overall model						7.69	13, 481	**<0**.**001**	0.150
(Constant)	88.699	9.44		9.39	**<0**.**001**				
Age	0.107	0.09	0.05	1.20	0.230				
Sex	−0.409	1.38	−0.02	−0.30	0.767				
BD	0.410	14.35	0.01	0.03	0.977				
SZ	−6.663	11.70	−0.25	−0.57	0.569				
**ICV**	0.014	0.01	0.20	2.48	**0**.**013**				
BD*ICV	−0.004	0.01	−0.20	−0.43	0.669				
SZ*ICV	−0.001	0.01	−0.06	−0.12	0.901				
ASP	−15.138	21.67	−0.43	−0.70	0.485				
ASP*ICV	0.009	0.01	0.39	0.66	0.512				
BD*ASP	4.803	36.29	0.08	0.13	0.895				
SZ*ASP	−79.300	36.25	−1.16	−2.19	**0**.**029**				
BD*ASP*ICV	0.000	0.02	0.01	0.02	0.985				
SZ*ASP*ICV	0.048	0.02	1.12	2.13	**0**.**034**				
**Verbal IQ**									
Overall model						4.41	13, 481	**<0**.**001**	0.082
(Constant)	88.193	10.06		8.77	**<0**.**001**				
Age	−0.071	0.10	−0.03	−0.75	0.454				
Sex	−1.789	1.47	−0.07	−1.22	0.225				
BD	22.12	15.29	0.72	1.45	0.149				
SZ	6.215	12.46	0.23	0.50	0.618				
**ICV**	0.015	0.01	0.21	2.56	**0**.**011**				
BD*ICV	−0.016	0.01	−0.82	−1.68	0.095				
SZ*ICV	−0.008	0.01	−0.49	−1.06	0.289				
ASP	9.714	23.08	0.27	0.42	0.674				
ASP*ICV	−0.007	0.01	−0.33	−0.53	0.597				
BD*ASP	−26.473	38.64	−0.43	−0.69	0.494				
SZ*ASP	−106.146	38.60	−1.51	−2.75	**0**.**006**				
BD*ASP*ICV	0.021	0.02	0.53	0.86	0.393				
SZ*ASP*ICV	0.065	0.02	1.48	2.72	**0**.**007**				
**Performance IQ**									
Overall model						9.79	13, 481	**<0**.**001**	0.188
(Constant)	91.343	9.63		9.48	**<0**.**001**				
**Age**	0.293	0.09	0.14	3.21	**0**.**001**				
Sex	1.009	1.41	0.04	0.72	0.475				
BD	−24.344	14.64	−0.77	−1.66	0.097				
SZ	−21.337	11.93	−0.78	−1.79	0.074				
ICV	0.009	0.01	0.13	1.62	0.106				
BD*ICV	0.011	0.01	0.56	1.21	0.226				
SZ*ICV	0.009	0.01	0.51	1.17	0.242				
ASP	−37.575	22.10	−1.01	−1.70	0.090				
ASP*ICV	0.024	0.01	1.03	1.76	0.079				
BD*ASP	34.675	37.01	0.55	0.94	0.349				
SZ*ASP	−34.906	36.97	−0.49	−0.94	0.346				
BD*ASP*ICV	−0.020	0.02	−0.50	−0.87	0.385				
SZ*ASP*ICV	0.020	0.02	0.44	0.86	0.391				

BD (bipolar disorder spectrum); SZ (schizophrenia spectrum); IQ (intelligence quotient); ICV (intracranial volume); ASP (birth asphyxia).

Bold values denote statistical significance at the *p* < 0.05 level.

#### Performance IQ

In the regression analysis, we found a significant main effect of age. There was no main effect of ASP across the whole sample, but a marginally significant interaction between ICV and ASP (Supplemental materials: online Supplementary Fig. 1*B*; online Supplementary Table 3).

## Discussion

ICV of adult patients with SZ who experienced ASP showed a significantly greater positive association with IQ compared to SZ patients without ASP, a relationship that was statistically significant but less pronounced in the other groups. We found verbal IQ, not performance IQ, was the subcomponent that was positively associated with ICV and ASP in the SZ group.

The results support our initial hypothesis that a greater positive relationship between ICV and IQ exists in the subgroup of patients with SZ who have a history of ASP, compared to ASP effects in BD and HC. The reason for this association with the SZ group can be due to a vulnerability towards hypoxic stress in individuals genetically predisposed to develop SZ (Ursini et al., 2018), which results in a diminished resilience to the ASP. Cognitive consequences of the ASP might also be specific to SZ because the genetic variants associated with SZ risk overlap with genetic variants for both ICV (Smeland et al., 2018) and intelligence, where most of the SZ risk alleles were associated with poorer cognitive performance and opposite to what was found with BD risk alleles (i.e. better cognitive performance) (Smeland et al., 2019).

The heterogeneity in the stronger correlation between ICV and IQ of patients with SZ and ASP might be due to the ASP complication in the MBRN being unspecific to the amount of neural harm present in the infant. The degree of neural harm due to ASP might be reflected in the greater degree of relationship between ICV and IQ (i.e. great harm in those with small-ICV-low-IQ and less harm in those with large-ICV-high-IQ), but so might the variations in the genetic risk for SZ development, rendering some participants more or less resilient to hypoxic insult. Future studies might address the relationship between genetic risk for SZ development and ASP.

Smaller ICV has been reported in adolescent offspring of SZ compared to BD and HC offspring, together with significant associations between ICV and IQ in the patient offspring, but not in the HC offspring (van Haren et al., 2020). In our study, ICV of patients with SZ was smaller compared to both patients with BD and HC, after adjusting for ASP, but the association between IQ and ICV did not differ between groups. IQ showed a consistent dependence on ICV in both adult patient groups (reported here) and adolescent offspring of patients with SZ and BD (van Haren et al., 2020). Our findings raise the possibility that the varying degrees of impeded brain growth and cognitive development in SZ may be caused by neural insult due to ASP. Further, a small ICV and low IQ might serve as neurodevelopmental markers of SZ, and because both ICV and IQ measures can be assessed early in development, they have the potential to be included in multivariate prediction tools aiming to assess SZ risk at an early stage.

ICV being of particular importance for IQ in SZ might be related to greater hypoxic insult at birth, but an incidence of ASP reported in the MBRN does not provide information on the severity (i.e. mild, moderate or severe). We did not find a difference in the prevalence of ASP between patient groups and the HC. ASP is a primary cause of newborn death and neurological impairment, worldwide. Cerebral palsy is the neurological sequelae of fetal hypoxia occurring in pregnancy or around childbirth, which causes abnormal development or damage to parts of the brain that control movement, balance and posture (Volpe, 2012). Even if hypoxic injury due to ASP did not present early with motor and learning impairments, a primary neurological insult might be unmasked with later neural maturation challenges during adolescence and manifest as psychosis and cognitive and subtle neurological impairments in early adulthood (Murray, Bhavsar, Tripoli, & Howes, 2017).

ASP was associated with ICV and was specifically related to verbal IQ in the SZ group, which might indicate that impaired language abilities observed in SZ are partly a consequence of ASP. Language impairment in SZ has long been recognized (Fraser, King, Thomas, & Kendell, 1986; King, Fraser, Thomas, & Kendell, 1990; Morice & Ingram, 1982) and considered a key diagnostic feature of SZ spectrum disorders (de Boer, Brederoo, Voppel, & Sommer, 2020; DeLisi, 2001; Kuperberg, 2010*a*, *b*). In fact, verbal IQ was the subcomponent that differentiated patients with SZ and BD and might be a primary indicator of disease development. The language function is generally specialized to the left hemisphere of the human brain, and several studies have found a reduction of leftward language lateralization in patients with SZ (Hahn et al., 2011; Loberg, Hugdahl, & Green, 1999; Ocklenburg, Westerhausen, Hirnstein, & Hugdahl, 2013; Rossell & Boundy, 2005). An ASP might be related to the failure of the brain to specialize in language function (Crow, 1997). Our findings give further support for low verbal intellectual abilities as one of the main areas of cognition implicated in SZ development.

Genetic risk for the development of SZ might hinder cellular resilience to hypoxia in the fetal brain (Murray et al., 2017). Ischemia–hypoxia response genes (set of genes that respond to ischemia, insufficient blood flow to provide adequate oxygenation, which leads to tissue hypoxia) showed a threefold enrichment for genes associated with SZ risk (Schmidt-Kastner, van Os, Esquivel, Steinbusch, & Rutten, 2012). In addition, a study found that severe obstetric complications interact with ischemia–hypoxia response genes and further increase the risk for SZ (Nicodemus et al., 2008). A reduced up-regulatory effect on *HILPDA* (hypoxia-inducible lipid droplet-associated) expression was found in SZ (Akkouh et al., 2020). The functional HILPDA protein is expressed only under hypoxic stress (Jiang et al., 2015), and the failure to sufficiently up-regulate *HILPDA* expression might represent a cellular deficiency in SZ that fails to protect fetal neurons from any level of hypoxic insult. There is a need for further studies to better understand the hypoxic cascade behind neural injury and advance neuroprotective interventions such as hypothermia (Gluckman et al., 2005), xenon gas (Thoresen, Hobbs, Wood, Chakkarapani, & Dingley, 2009) and oxytocin (Boksa, Zhang, & Nouel, 2015), in efforts to decrease the risk for SZ development.

Strengths of this study were the use of the Medical Birth Registry of Norway, as a prospective study it is possible to establish a causal relationship between ASP and individuals who later develop SZ. This study provides evidence that ASP and ICV impact verbal IQ in SZ, which is currently missing in the field.

A limitation might be the FreeSurfer estimation of the ICV. From a T1-weighted scan, accurate measurement of the ICV is difficult since the signal intensities of cerebrospinal fluid and skull are not separable. FreeSurfer's registration-based estimates might be less accurate than segmentation-based estimates of ICV (Ma et al., 2019). Whereas it is most common to use IQ <70 as an exclusion criterion in studies of cognition in SZ, we included participants with IQ <70, which may have altered group proportions and means. However, in doing so better reflects patients with low intellectual abilities potentially attributed to ASP in the SZ population (Wells et al., 2015; Wortinger et al., 2019). A limitation of this study might also be the lower percentage of ASP + SZ patients with MRI data in our sample. We found ASP to be more prevalent in the BD group than the SZ group, which is unexpected if SZ risk predisposes patients to early insults around birth. Since OCs in SZ were associated with lower IQ (Wortinger et al., 2019), it could be that subgroups of SZ patients with lower IQ are less likely to participate in additional MRI studies. Furthermore, ASP + SZ patients might have had obvious organic pathology/brain lesions in their MR images, which would have excluded them from this study. With the performance IQ analysis, we did not find statistically significant main effects or interactions with ASP, ICV and group variables. However, the trend-level ASP and ICV interaction in the model suggests ASP effects on ICV might have a general impact on performance IQ and this relationship may not be disease specific. Future studies with larger samples are needed to clarify and replicate these results.

Small sample size in the BD group might be a limitation in this study, which may lead to difficulty in finding an interaction effect between ASP and ICV on IQ (false negative). Even so, we found a greater prevalence of ASP in the BD group compared the SZ group, and previously reported smaller ICV in BD patients with ASP compared to BD patients without ASP (Wortinger et al., 2020). Yet, we did not find an interaction between ASP and ICV on IQ in BD. Thus, despite the non-significant statistical difference, the findings suggest that IQ is less dependent on ICV in BD, and that BD is more resilient to hypoxic injury than SZ.

## Conclusion

We found a significantly stronger relationship between intracranial volume and IQ, specifically verbal IQ, in patients with SZ who had experienced perinatal or birth asphyxia compared to SZ patients who did not. This moderation by ASP was not demonstrated in the patients with BD or in HC. The greater positive association between ICV and IQ in patients with SZ who had experienced ASP might indicate abnormal neurodevelopment. Our findings give support for ICV together with verbal intellectual abilities as clinically relevant markers that can be added to prediction tools to enhance evaluations of risk in SZ development. Improved pre- and perinatal care and ready neuroprotective interventions for vulnerable pregnancies may lessen interactive risks caused by ASP.

## Supplementary material

For supplementary material accompanying this paper visit https://doi.org/10.1017/S0033291721000489. click here to view supplementary material
