# Supplementary material for: Significant association between intracranial volume and verbal intellectual abilities in patients with schizophrenia and a history of birth asphyxia
Source: Psychol Med. 2021 Mar 10;52(15):3698–707. doi: 10.1017/S0033291721000489 (PMC9772907; doi:10.1017/S0033291721000489)
Supplement: Supplementary file 1 [file S0033291721000489sup001.docx]

**Significant association between intracranial volume and verbal intellectual abilities in patients with schizophrenia and a history of birth asphyxia**

Laura Anne Wortinger * ^1, 2^, Kjetil Nordbø Jørgensen ^1, 2^, Claudia Barth ^2^, Stener Nerland ^1, 2^, Runar Elle Smelror ^1, 2^, Anja Vaskinn ^2, 3^, Torill Ueland ^2, 3, 4^, Ole A. Andreassen ^2, 3^ and Ingrid Agartz ^1, 2, 5^

^1^Department of Psychiatric Research, Diakonhjemmet Hospital, Oslo, Norway

^2^ NORMENT, Institute of Clinical Medicine, University of Oslo, Oslo, Norway

^3^NORMENT, Division of Mental Health and Addiction, Oslo University Hospital, Oslo, Norway

^4^Department of Psychology, University of Oslo, Oslo, Norway

^5^Centre for Psychiatric Research, Department of Clinical Neuroscience, Karolinska Institute, Stockholm, Sweden

*To whom correspondence should be addressed: Dr. Laura Anne Wortinger, Department of Psychiatric Research, Diakonhjemmet Hospital, Postbox 23 Vinderen, 0319, Oslo, Norway; e-mail: [l.a.w.bakke@medisin.uio.no](mailto:l.a.w.bakke@medisin.uio.no)

**Supplemental materials**

**A**

B

**Figure 1, A.** In the SZ group, the relationship between ICV and IQ was significantly greater in the presence of ASP compared to when ASP was not present, which was not found in the other groups. **B.** For performance IQ, there was no main effect of ASP across the whole sample, but a marginally significant interaction between ICV and ASP. IQ (intelligence quotient); ICV (intracranial volume); ASP (birth asphyxia).
